# Radiotherapy in the Caribbean: a spotlight on the human resource and equipment challenges among CARICOM nations

**DOI:** 10.1186/s12960-020-00489-5

**Published:** 2020-07-17

**Authors:** Kellie Alleyne-Mike, Pearse Sylvester, Vladimir Henderson-Suite, Thana Mohoyodeen

**Affiliations:** 1 National Radiotherapy Centre, 112 Western Main Road, St. James, Port of Spain, Trinidad and Tobago; 2 Port of Spain General Hospital, 61 Charlotte Street, Port of Spain, Trinidad and Tobago

**Keywords:** CARICOM, Caribbean, Radiation Oncology, Radiotherapy, Workforce

## Abstract

**Background:**

There is limited data on access to radiotherapy services for CARICOM nations.

**Methods:**

This was a descriptive mixed-methods observational study which used data collected via survey from staff working in Radiation Oncology in 14 CARICOM countries. Benchmark recommendations from the International Atomic Energy Agency were compared to existing numbers. The Directory of Radiotherapy Centers, World Bank, and Global Cancer Observatory databases were all accessed to provide information on radiotherapy machines in the region, population statistics, and cancer incidence data respectively. Both population and cancer incidence-based analyses were undertaken to facilitate an exhaustive review.

**Results:**

Radiotherapy machines were present in only 50% of the countries. Brachytherapy services were performed in only six countries (42.9%).

There were a total of 15 external beam machines, 22 radiation oncologists, 22 medical physicists, and 60 radiation therapists across all nations.

Utilizing patient-based data, the requirement for machines, radiation oncologists, medical physicists, and radiation therapists was 40, 66, 44, and 106, respectively. Only four (28.6%) countries had sufficient radiation oncologists. Five (35.7%) countries had enough medical physicists and radiation therapists.

Utilizing population-based data, the necessary number of machines, radiation oncologists, and medical physicists was 105, 186, and 96 respectively. Only one county (7.1%) had an adequate number of radiation oncologists. The number of medical physicists was sufficient in just three countries (21.4%). There were no International Atomic Energy Agency population guidelines for assessing radiation therapists.

A lower economic index was associated with a larger patient/population to machine ratio. Consequentially, Haiti had the most significant challenge with staffing and equipment requirements, when compared to all other countries, regardless of the evaluative criteria. Depending on the mode of assessment, Haiti’s individual needs accounted for 37.5% (patient-based) to 59.0% (population-based) of required machines, 40.1% (patient-based) to 59.7% (population-based) of needed radiation oncologists, 38.6% (patient-based) to 58.3% (population-based) of medical physicists, and 42.5% (patient-based) of radiation therapists.

**Conclusion:**

There are severe deficiencies in radiotherapy services among CARICOM nations. Regardless of the method of comparative analysis, the current allocation of equipment and staffing scarcely meets 50% of regional requirements.

## Background

With cancer incidence rising globally, developing countries can be particularly susceptible due to potential resource limitations [1]. The Caribbean is one such region in which access to services is not only limited but where specific variations exist across the entire grouping of islands due to differences in the socio-economic background of the countries [2].

Access to radiation therapy (RT) is a significant challenge with deficiencies in equipment and staffing being identified globally [3, 4]. The lack of specialists hinders development as these facilities need to be operated by specially trained staff. These include radiation oncologists, medical physicists, and radiation therapists as well as other critical support staff such as radiation oncology nurses, radiologists, dosimetrists, and other allied specialties that support the continuum of care.

In many studies, shared analyses of regional data have been submitted collectively for the Caribbean and Latin America, but in reality, each area is unique. Given the limited individual data on the Caribbean, it can be challenging to separate the specific situation in the Caribbean from the rest of the region. The Caribbean Community (CARICOM) consists of an organization of member states whose goal it is to foster cooperation among the island nations. This study focuses on the full member states. It aims to identify the existing services for therapeutic radiation available in the region and analyze their adequacy compared to established recommendations for essential service delivery.

## Methods

This study is a descriptive observational study with a focus on the CARICOM region and its full member states. It was conducted from February 2018 to March 2019. Fourteen CARICOM countries including Antigua and Barbuda, The Bahamas, Barbados, Belize, Dominica, Grenada, Guyana, Haiti, Jamaica, St. Kitts and Nevis, St. Lucia, St. Vincent and the Grenadines, Suriname, and Trinidad and Tobago were evaluated. Montserrat, a British overseas territory, was the only full member country which was excluded. In Montserrat, patients can access care through the United Kingdom, and therefore, this country does not have the same limitations as exists with the other areas identified. It thus could not be fairly compared to the remaining countries.

The study utilized a mixed-methods approach with data combined from multiple sources including a questionnaire for staffing information, the Directory of Radiotherapy Centers (DIRAC) database for therapeutic equipment tallies, population data from the World Bank database, and Global Cancer Observatory Database (GLOBOCAN) to guide estimations on the annual number of newly diagnosed cancer patients [5–7].

All assessed employees needed to be actively practicing. Population and patient data were used to compare existing staffing and equipment resources to International Atomic Energy Agency (IAEA) recommendations [8, 9]. These guidelines stated that the requirement for radiation oncologists should be one per 100 000 inhabitants or one per 250 patients per year. The number of physicists needed to provide radiation therapy services was one per 200 000 persons or one per 400 patients per year. IAEA population-based recommendations were not available for radiation therapists (RTTs); however, patient-based guidelines were used [8]. The recommendation was for 150 patients per year per RTT.

The GLOBOCAN information was utilized along with the IAEA estimations for the Caribbean region, which suggested that 55% of all cancer patients will require radiation therapy [10]. Given that GLOBOCAN incidence statistics were not available for all countries, a crude cancer incidence rate was used to estimate the number of new cases for the countries lacking data. This rate was calculated using the average of new cancer cases per population based on the nine CARICOM countries with known cancer incidence data. The exception to this would have been instances where no radiotherapy machines were available. Consequently, the staffing needs could be considered as 100% deficient because professionals needed to be in employment at the time of the assessment. This could not be possible without the equipment available on site.

Data regarding the number of radiotherapy machines and the availability of brachytherapy services available in a given country were taken from the Directory of Radiotherapy Centers (DIRAC) [5]. In the case of external beam machines, it was recommended that one machine was required per 180 000 inhabitants or for every 450 patients diagnosed per year [8, 9]. IAEA guidelines for brachytherapy equipment were not published. All values were rounded to the nearest integer except values which were less than one. The crude cancer incidence rate was rounded to three significant digits.

## Results

Data for all categories were collected from 14 Caribbean countries.

Table 1 displays the number of radiotherapy services existing in the countries identified compared to the recommended number of machines according to IAEA guidelines (population and patient-based criteria). The names of the facilities were included in addition to whether public or private services were offered. The total existing number of megavoltage machines was 15. The overall numbers required according to population and patient-based criteria were 105 and 40, respectively. Only 50% of the countries studied had megavoltage units. Antigua and Barbuda is the only country that satisfies recommendations, regardless of population or patient-based criteria. All other countries (92.9 %) had less than the needed amount in at least one type of guidance, with Haiti having the most significant challenge. Jamaica does not satisfy population or patient-based criteria in spite of having the most machines. Belize, Dominica, Grenada, Haiti, St. Kitts and Nevis, St Lucia, and St Vincent and the Grenadines have no radiotherapy machines; hence, they cannot meet any criteria. The Bahamas, Guyana, and Trinidad and Tobago satisfied the patient-based criteria but not the population-based criteria. Suriname surpasses patient-based guidelines but does not meet population-based criteria.

**Table 1:** Number of radiotherapy services per country compared to the recommended number of machines

Country	Population (thousands)^**1**^	No. of patients (GLOBOCAN data)	Institutions	Existing external beam radiation therapy machines			Recommended radiotherapy machines (population based)^**2**^	Recommended radiotherapy machines (patient based)^**3**^
			Public/private affiliation	Cobalt-60	LINAC	Total		
**Antigua and Barbuda**	96	198*	The Cancer Centre Eastern Caribbean (private)	-	1	1	< 1	< 1*
**The Bahamas**	386	933	The Cancer Centre Bahamas (private)	-	1	1	2	1
**Barbados**	287	1245	Queen Elizabeth Hospital (public)	1	-	1	2	2
**Belize**	383	358	-	-	-	0	2	< 1
**Dominica**	72	148*	-	-	-	0	< 1	< 1*
**Grenada**	111	230*	-	-	-	0	< 1	< 1*
**Guyana**	779	751	Cancer Institute of Guyana (private)	-	1	1	4	< 1
**Haiti**	11 123	12 366	-	-	-	0	62	15
**Jamaica**	2935	7348	Cornwall Centre for Cancer Treatment (public) Kingston Public Hospital (public) Radiation Oncology Centre of Jamaica (private) National Cancer Treatment Centre, St. Joseph’s Hospital (public)	2	3	5	16	9
**St. Kitts and Nevis**	52	108*	-	-	-	0	< 1	1*
**St. Lucia**	182	376	-	-	-	0	1	< 1
**St. Vincent and the Grenadines**	110	227*	-	-	-	0	< 1	< 1*
**Suriname**	576	1042	Radiotherapeutic Centre Suriname, Academic Hospital Paramaribo (public)	-	2	2	3	1
**Trinidad and Tobago**	1390	3369	National Radiotherapy Centre, St, James Medical Complex (public) Brian Lara Cancer Treatment Centre (private) Southern Medical Clinic (private)	1	3	4	8	4
**All countries**	**18 482**	**28 699**		**4**	**11**	**15**	**105**	**40**

*Using average crude incidence ratio (new cases/population) of the nine CARICOM nations with known incidence data from GLOBOCAN = 0.002 06 (95% CI = 0.000 82)

^1^Based on World Bank population data 2019 revision [6]

^2^One machine per 180 000 inhabitants

^3^One per 450 patients/year (using radiation utilization rate of 55% and GLOBOCAN data) [7]

In Table 2, the presence of brachytherapy services is displayed. Brachytherapy services were performed in six countries (42.9%) but absent in the remaining eight (57.1%) nations.

**Table 2:** The availability of brachytherapy services in each of the islands

Country	Brachytherapy available
Antigua and Barbuda	No
The Bahamas	Yes
Barbados	Yes
Belize	No
Dominica	No
Grenada	No
Guyana	Yes
Haiti	No
Jamaica	Yes
St. Kitts and Nevis	No
St. Lucia	No
St. Vincent and the Grenadines	No
Suriname	Yes
Trinidad and Tobago	Yes

Tables 3, 4, and 5 focused on the number of skilled staff available in the disciplines of radiation oncology, medical physics, and radiation therapy. The figures portray the disparity between actual numbers and those recommended by the IAEA. The total numbers of existing staff with respect to radiation oncologists, medical physicists, and radiation therapists were 22, 22, and 60, respectively. Belize, Dominica, Grenada, Haiti, St. Kitts and Nevis, St Lucia, and St Vincent and the Grenadines have no radiotherapy machines; hence, they cannot meet the criteria for any staffing sector. Haiti had the most significant challenge due to the relatively large population size and the absence of any external beam radiation services.

**Table 3:** Number of radiation oncologists per country compared to the numbers recommended by the IAEA

Country	Existing	Recommended^**1**^ (population based)	Absolute difference (population based)	Recommended^**2**^ (patient based)	Absolute difference (patient based)
**Antigua and Barbuda**	3	< 1	2	< 1*	2
**The Bahamas**	2	4	− 2	2	0
**Barbados**	1	3	− 2	3	− 2
**Belize**	0; NRM	4	− 4	< 1	− 1
**Dominica**	0; NRM	< 1	− 1	< 1*	− 1
**Grenada**	0; NRM	1	− 1	< 1*	− 1
**Guyana**	1	8	− 7	2	− 1
**Haiti**	0;NRM	111	− 111	27	− 27
**Jamaica**	6	29	− 23	16	− 10
**St. Kitts and Nevis**	0; NRM	< 1	− 1	< 1*	− 1
**St. Lucia**	0; NRM	2	− 2	< 1	− 1
**St. Vincent and the Grenadines**	0; NRM	1	− 1	< 1*	− 1
**Suriname**	2	6	− 4	2	0
**Trinidad and Tobago**	7	14	− 7	7	0
**All countries**	22	186	− 164	66	− 44

*NRM* no radiotherapy machine

*Using average crude incidence ratio (new cases/population) of the nine CARICOM nations with known incidence data from GLOBOCAN = 0.002 06 (95% CI = 0.000 82)

^1^One per 100 000 inhabitants (per population) and using World Bank population data 2019 revision [6]

^2^One per 250 patients/year (using radiation utilization rate of 55% and GLOBOCAN data) [7]

**Table 4:** Number of medical physicists per country compared to the numbers recommended by the IAEA

Country	Existing	Recommended^**1**^ (per population)	Absolute difference (per population)	Recommended^**2**^ (patient based)	Absolute difference (patient based)
**Antigua and Barbuda**	2	< 1	1	< 1*	1
**The Bahamas**	2	2	0	1	1
**Barbados**	1	1	0	2	− 1
**Belize**	0; NRM	2	− 2	< 1	− 1
**Dominica**	0; NRM	< 1	− 1	< 1*	− 1
**Grenada**	0; NRM	< 1	− 1	< 1*	− 1
**Guyana**	1	4	− 3	1	0
**Haiti**	0; NRM	56	− 56	17	− 17
**Jamaica**	9	15	− 6	10	− 1
**St. Kitts and Nevis**	0; NRM	< 1	− 1	< 1*	− 1
**St. Lucia**	0; NRM	< 1	− 1	< 1	− 1
**St. Vincent and the Grenadines**	0; NRM	< 1	− 1	< 1*	− 1
**Suriname**	1	3	− 2	1	0
**Trinidad and Tobago**	6	7	− 1	5	1
**All countries**	22	96	− 74	44	− 22

*NRM* no radiotherapy machine

*Using average crude incidence ratio (new cases/population) of the nine CARICOM nations with known incidence data from GLOBOCAN = 0.00206 (95% CI = 0.00082)

^1^One per 200 000 persons (per population) and using World Bank population data 2019 revision

^2^One per 400 patients/year (using radiation utilization rate of 55% and GLOBOCAN data)

**Table 5:** Table of radiotherapy technologist (RTT) staffing (150 patients/year/RTT)

Country	Assuming 55% of patients require radiotherapy	Recommended staffing	No. in country	Absolute difference
**Antigua and Barbuda**	109*	< 1	3	2
**The Bahamas**	513	3	3	0
**Barbados**	685	5	5	0
**Belize**	197; NRM	1	0	− 1
**Dominica**	81*; NRM	< 1	0	− 1
**Grenada**	127*; NRM	< 1	0	− 1
**Guyana**	413	3	1	− 2
**Haiti**	6801; NRM	45	0	− 45
**Jamaica**	4041	27	13	− 14
**St. Kitts and Nevis**	59*; NRM	< 1	0	− 1
**St. Lucia**	207; NRM	1	0	− 1
**St. Vincent and the Grenadines**	125*; NRM	< 1	0	− 1
**Suriname**	573	4	11	7
**Trinidad and Tobago**	1853	12	24	12
**All countries**	15 784	106	60	− 46

All values were rounded to the nearest integers for total calculations

*NRM* no radiotherapy machine

*Using average crude incidence ratio (new cases/population) of the nine CARICOM nations with known incidence data from GLOBOCAN = 0.00206 (95% CI = 0.00082)

In Table 3, seven countries had radiation oncologists working in the field. The recommended totals for all nations by population- and patient-based standards were 186 and 66, respectively. Antigua and Barbuda is the only country (accounting for 7.1% of the studied population) that met IAEA requirements for clinician staffing by all criteria. No other country met population-based criteria. Barbados, Guyana, and Jamaica did not meet any criteria. The Bahamas, Suriname, and Trinidad and Tobago met the patient-based criteria only.

In Table 4, the recommended totals for all nations by population- and patient-based standards were 96 and 44, respectively. Only two of the countries (14.3%), Antigua and Barbuda and The Bahamas, had the correct quota of medical physicists available by all criteria. Jamaica did not meet any staffing criteria. Barbados met the population-based criteria while Suriname, Trinidad and Tobago, and Guyana met patient-based criteria.

Table 5 shows the distribution of RTT staffing. The recommended total for all nations by patient-based criteria was 106. The Bahamas, Barbados, Suriname, Antigua and Barbuda, and Trinidad and Tobago (35.7%) were the only countries to satisfy the criteria. The last three countries surpassed the staff requirements by at least 50%.

Table 6 shows the distribution of skilled staff across institutions based on public or private service delivery. The majority of all staffing categories worked in the public sector. The medical physics staff however was almost the same across both public and private facilities.

**Table 6:** The distribution of staff in the public and private sector for each discipline per country

Radiation Oncology Workforce		
**Clinician**		
**Country**	**Public**	**Private**
**Antigua and Barbuda**	-	3*^(part time)^
**The Bahamas**	-	2
**Barbados**	1	-
**Guyana**	-	1
**Jamaica**	5	1
**Suriname**	2	-
**Trinidad and Tobago**	4	3
**Total**	12 (63.2%)	7 (36.8%)
**Medical physicist**		
**Country**	**Public**	**Private**
**Antigua and Barbuda**	-	2
**The Bahamas**	-	2
**Barbados**	1	-
**Guyana**	-	1
**Jamaica**	7	2
**Suriname**	1	-
**Trinidad and Tobago**	4	2
**Total**	13 (56.5%)	10 (43.5%)
**Radiation therapist**		
**Country**	**Public**	**Private**
**Antigua and Barbuda**	-	3
**The Bahamas**	-	3
**Barbados**	5	0
**Guyana**	-	1
**Jamaica**	10	3
**Suriname**	11	0
**Trinidad and Tobago**	16	8
**Total**	42 (68.9%)	19 (31.1%)

Table 7 demonstrates that a decrease in the economic index was associated with a decrease in the availability of radiation therapy services (machines). Cumulatively middle-income countries in this study had twice the number of individuals being treated on almost the same number of machines as in the high-income countries (eight external beam machines versus seven, respectively). Haiti was the only country categorized as low income and did not have any machines available.

**Table 7:** Megavoltage machines per population size and proportion of nations with megavoltage machines and brachytherapy services based on economic index grouping

Country grouped by economic index^**1**^	Population per country (thousands)^**2**^	No. of patients (GLOBOCAN data)	Total population (thousands)^**2**^	Total incident population	No. of megavoltage machines	Persons per megavoltage machines	Estimated patients requiring radiotherapy per megavoltage machine^**3**^	Proportion with at least one megavoltage machine	No. of countries with brachytherapy services	Proportion with brachytherapy services
**High**										
**Antigua and Barbuda**	96	198*	2211	5853	7	315 838	460	80%	3	60%
**The Bahamas**	386	933								
**Barbados**	287	1245								
**St. Kitts and Nevis**	52	108*								
**Trinidad and Tobago**	1390	3369								
**Upper middle**										
**Belize**	383	358	5148	10 480	8	643 512	721	38%	3	38%
**Dominica**	72	148*								
**Grenada**	111	230*								
**Guyana**	779	751								
**Jamaica**	2935	7348								
**St. Lucia**	182	376								
**St. Vincent and the Grenadines**	110	227*								
**Suriname**	576	1042								
**Low**										
**Haiti**	11 123	12 366	11 123	12 366	0	Not evaluable	Not evaluable	0%	0	0%

*Using average crude incidence ratio (new cases/population) of the nine CARICOM nations with known incidence data from GLOBOCAN = 0.00206 (95% CI = 0.000 82)

^1^According to the World Bank Gross National Income data 2019 revision (Criteria ranges in US dollars: low = $1025 or less; upper middle = $3996 to $12 375; high = $12 376 or more) [11]

^2^Based on World Bank population data 2019 revision

^3^Using radiation utilization rate of 55% and GLOBOCAN data

Haiti represents 43.1% of the total patient population studied. Haiti’s individual needs proportionally accounted for 37.5% (patient-based) to 60.2% (population-based) of required machines, 40.1% (patient-based) to 59.7% (population-based) of needed radiation oncologists, 38.6% (patient-based) to 58.3% (population-based) of medical physicists, and 42.5% (patient-based) of radiation therapists.

## Discussion

Radiation therapy continues to be one of the three main pillars for cancer treatment (along with surgery and chemotherapy), with approximately 50% of patients in developed countries requiring treatment at some period during their disease [8]. This estimated percentage is increased for LMIC (55% in the Caribbean) due to the types of cancers that are prevalent, and patients being diagnosed at later stages, where surgery is a limited option [10]. Its utility can also extend beyond the treatment of malignancies to encompass benign conditions. Still, access to radiation therapy can be overlooked despite the significant and growing need. The leading causes of the problem remain the same in lower- and middle-income countries (LMIC) across regions, namely, lack of equipment, insufficient staff, and a dearth of information to inform decisions [12]. The equipment required for delivery of the service is also associated with significant start-up and maintenance costs. In a study published by Bishr et al., there were only three countries in the African and Latin American regions, which were able to meet IAEA standards for population-based megavoltage accessibility [13]. Zubizarreta et al. identified similar deficiencies in other areas globally [14].

Fifty percent of the CARICOM countries analyzed in this study did not have radiotherapy machines (Table 1). These countries included Belize, Dominica, Grenada, Haiti, St. Kitts and Nevis, St. Lucia, and St. Vincent and the Grenadines. They accounted for approximately 65% of the total population of all the islands together based on World Bank Data (note that Haiti alone accounts for 60%) [6]. In Belize, patients needing this service sought treatment privately in Mexico with occasional support from the Cancer Society. In Haiti, patients who could afford private care would try to access this service in the Dominican Republic. Patients from the other islands would try to obtain services privately in Antigua and Barbuda, Barbados, or Trinidad and Tobago primarily. Among those with machines capable of delivering external beam radiation therapy, only Antigua and Barbuda had sufficient machines to meet both population- and patient-based requirements. Therefore, the majority of countries (92.9 %) did not have the required capacity by at least one type of criteria.

Based on each country’s individual patient criteria, ten countries would require at least one external beam machine each. Seven of these countries had estimations of less than one machine per country. This suggests that fully outfitting each of those seven countries would create a scenario in which machines would be underutilized. However, when the cumulative patient population (4371) of all seven was assessed together, this value actually favored the need for all of these ten machines (9.7 machines). Thus, acquisition and sharing of resources among the ten countries would effectively meet the needs of the collective. The remaining four countries needed two machines or more and collectively required 30 machines (accounting for 75% of the equipment required). Two of these countries met their patient-based criteria while the other two did not.

Linear accelerators have also gradually been replacing cobalt radiotherapy machines and are capable of offering more precision in targeting treatment. These machines are also capable of providing therapy with both high energy X-rays for deep tumors and electrons for superficial tumors. Only one of the seven countries with machines (Barbados) did not possess a linear accelerator. Two of the remaining six (Jamaica and Trinidad and Tobago) both still had cobalt units in addition to their linear accelerators. However, the number of linear accelerators surpassed cobalt machines and the former were more recently acquired, suggesting a trend towards decreased reliance on cobalt machines and the inherent limitations such as decaying source activity, limitations in reducing toxicity to organs at risk, and the inability to deliver superficial treatments.

In this study, the presence of brachytherapy services was also documented. Cervical and endometrial brachytherapy are considered an essential component of the curative radio-therapeutic management of most stages of cervical cancer and some stages of endometrial cancer. There are, therefore, adverse survival implications in its absence. GLOBOCAN data also shows that cervical cancer remains one of the top three cancers for women in CARICOM countries [7]. More than half of the studied nations, accounting for 57.1% of the grouping, did not have brachytherapy services (Table 2). Antigua and Barbuda, which possessed adequate external beam radiotherapy services, did not have access to brachytherapy services on that island. Patients requiring this treatment tended to access this privately through The Bahamas, Barbados, or Trinidad and Tobago primarily.

Megavoltage machines are expensive and require highly specialized staff, and maintenance is also costly for developing countries. In spite of this, it has been shown that the patient lives and costs saved in providing timely and adequate radiation therapy will greatly outweigh what is expended in offering the service [15, 16]. Yap and colleagues estimated that in LMIC, as much as 1.3 million people would derive a local control benefit by 2035 if the demand for radiotherapy was met. They also calculated that an additional 615 000 patients would have a survival benefit [17]. Other countries in the region, such as the Dominican Republic and Puerto Rico, have a more significant concentration of radiotherapy machines in their countries [5]. This may present an opportunity for CARICOM nations to share resources with these places. It will, however, require government to government discussions and agreements. There will also be additional hurdles to overcome, such as the geographic distance between islands and language barriers. Haiti, in particular, is a country where a significant need has been identified, and with its close proximity to the Dominican Republic, a partnership seems logical.

The lack of equipment and infrastructure is not the only challenge. These facilities need to be staffed with skilled personnel. The category of specialist doctors trained and working in medical oncology, radiation oncology, and hematology in the Caribbean is also limited, according to Alleyne-Mike, with its key personnel in high demand [18]. That paper, while focusing on the clinician workforce, did not include allied staff (e.g., radiation therapists and medical physicists) who are also necessary for providing the full complement of care. In the current study, only Antigua and Barbuda had an adequate number of radiation oncologists to satisfy all recommended criteria. Though it should be noted, not all of these oncologists were based at the facility as some offered part-time service between Antigua and Barbuda and the Bahamas due to a shared institutional partnership. The remaining 13 countries (92.9%) did not have the requisite quantity of these specialists to satisfy both criteria (Table 3). The Bahamas and Trinidad and Tobago had at least half of the necessary staffing according to population criteria, and both these countries along with Suriname met patient-based recommended numbers. Still, in all other countries, the staff deficit was at least 50%. Multiple studies have shown a correlation between radiation oncology density and survival, with a low density of radiation oncologists being associated with increased mortality [19–21]. Thus, these insufficient numbers are concerning with obvious potential for adverse repercussions on patient survival.

A similar staff challenge was noted when reviewing medical physicists. In the current study, only two countries (14.3%) had adequate staff numbers of medical physicists (Table 4) using both patient- and population-based criteria. These countries were Antigua and Barbuda and The Bahamas. Barbados met the population-based criteria while Suriname, Trinidad and Tobago, and Guyana met patient-based criteria only. The remaining countries (57.1%) had inadequate staffing based on both criteria. This shortage was also seen in a study published by Tsapaki and co-workers, which also used a questionnaire format [22]. The paper looked at the medical physicist workforce, concluding that the global availability of this skill did not match clinical needs. Latin America, the Caribbean, and Africa were identified as having the highest deficit. The study also projected that by 2035 the global need for medical physicists would be double the amount available today.

The number of radiation therapists was appropriate in five countries (35.7%) by all criteria (Table 5). Suriname and Trinidad and Tobago appeared to have surplus staffing. However, therapists in these facilities also have additional responsibilities associated with computed tomography simulation or brachytherapy services apart from work needed at the radiotherapy machines as previously mentioned, and for which the additional staffing would be assigned to these supplemental duties. Some of the radiation therapists were trained to perform dosimetry as an additional work responsibility, and thus, they similarly had divided tasks.

The authors Zubizaretta et al. determined that over 43 000 people were needed to fully equip the global radiation treatment workforce [3]. This workforce should ideally include dosimetrists, who were not quantified in the study. However, many of the institutions indicated that their medical physicists or RTTs fulfilled the function of dosimetrists. This dual functionality is an asset and necessary in regions with limited trained staff. It, however, poses the problem of further increasing the workload of the treating medical physicist. In Trinidad and Tobago, the medical physicists at one public institution also served as the radiation safety officers for at least three additional general hospitals, multiple local health centers, and other public authorities with radiation equipment outside the arena of health. Thus, their skill was not concentrated at the medical facility and associated primarily with therapeutics but was diluted across multiple other institutions. A similar parallel was highlighted in the paper by Alleyne-Mike, in which clinicians offering radiation oncology services in the Caribbean would have entered training programs with a Clinical Oncology curriculum allowing the specialist to be qualified in both radiation and medical oncology [18]. This is extremely valuable when medical and radiation oncologists are both in short supply. However, it should be noted that the IAEA recommendations were based on a radiation oncologist who is devoted solely to providing radiation treatments. Oncologists having to divide their time between a medical oncology clinic and a full radiation oncology workload will result in further limitations in access to both of those services. Therefore, the existing numbers of radiation oncologists noted in Table 3 are unable to reflect this identified gap, and it was not possible to estimate the percentage of the clinical oncologist’s time that is allocated to radiation oncology in each specific center. Consequently, the instances where some of the country estimates have suggested over-staffing may be misleading given these shared responsibilities. In addition, some country estimates recommend a sole oncologist or physicist. Operating a facility with a single radiation oncologist, medical physicist, or radiation therapist is inherently problematic as it makes no provision for leave coverage (unexpected or planned). Radiation safety quality and control procedures also often require second checks by similarly qualified staff, and this cannot be facilitated with a single person occupying the role. If we take this into account, then the medical physicist staff requirement will no longer be met in countries such as Guyana, Suriname, and Barbados. Therefore, given all of the above, the staff deficits for radiation oncologists and medical physicists (Tables 3 and 4) that were found in this study may be higher.

The IAEA has been playing a pivotal role in supporting LMIC in the development of their radiotherapy services through collaborations with organizations such as the World Health Organization and local governments. These collaborations could also be beneficial to Caribbean nations [23].

Table 6 shows the distribution of workers in the public and private sectors for each discipline. It is important to note that the collective staff assessments shown were applied to staff regardless of whether they were working in the public or the private sector. However, due to the significant costs associated with oncology treatment, the majority of the population tends to access care in the public sector in countries where this is a possibility. Thus, here again the apparent surplus of staff suggested in some of the tables may be misleading as 31.1% (RTTs), 36.8% (oncologists), and 43.5% (medical physicists) worked in the private sector. Given the high costs of care, public facilities logically are more likely to treat higher patient numbers, yet only Suriname and Barbados offered access to treatment solely in the public sector. Public run facilities were not identified in Antigua and Barbuda, Guyana, or The Bahamas. In Antigua and Barbuda, patients who were referred for treatment through the government were treated at a reduced fee. In Jamaica and Trinidad and Tobago, both public and private radiation therapy services were offered. A proportion of public patients in Trinidad and Tobago are referred to the private facilities through public/private partnerships.

Planning radiotherapy services requires country-specific data on which to base estimations and make recommendations, but information for the Caribbean is limited. Many Caribbean countries do not yet have a fully operational cancer registry [24]. Cancer incidence, mortality, and prevalence data are vital for policy development, infrastructure and workforce planning, cancer prevention, risk reduction, and cancer screening programs.

Currently, there is a low research output, and also limited data, from the region. Aggarwal and colleagues found that most of the research publications on radiation therapy emanated from the United States of America, Japan, and Germany [25]. The paper focused on phase one, two, and three studies. Only four LMIC were identified amongst the top 25 countries, and none of these were located in the Caribbean or Central America. Other studies have also suggested that medical physicists and radiation therapists are more likely to stay in their profession if they were involved in research [26, 27]. For the studied region, retention of staff can be a concern. These individuals can be influenced to migrate to countries and institutions where the financial remuneration is greater and where the prospects of working with more sophisticated equipment are higher, thus increasing their potential for skill retention and enhancement. Local training programs for radiation therapy technologists and medical physicists are in existence in some of the countries, and these degrees are recognized within the Caribbean. The existence of these programs is a positive step to both increase capacity and also retain staff with degrees recognized only loco-regionally. There are, however, no radiation oncology training programs in the Caribbean, and therefore, clinicians need to venture externally to obtain the needed knowledge. Clinicians interested in pursuing the field must compete with international candidates for posts which in many cases must be first preferentially allocated to the nationals in the respective countries offering the program.

The impact on patient care and survival cannot be ignored as patients who are not treated optimally can progress to advanced stages of the disease. These patients require long-term palliative care with frequent hospital visits, often resulting in repeat ward admissions and all the associated costs. Palliative radiotherapy also can be a cost-effective treatment in the management of symptoms due to advanced and metastatic disease [28]. We must also consider the long-term costs for medications to control pain and procedures geared at hemostasis, to name a few. In an article by Konski and co-workers, the authors pointed out that the cost associated with a prolonged course of narcotics was higher than that of a single fraction of palliative radiotherapy (where clinically applicable) [29].

Table 7 shows countries grouped according to their ranking by economic index. The total number of inhabitants in middle-income countries was approximately double that of the higher-income countries. Yet, the total number of external beam machines available was almost the same. This also held true when patient incident data was used with higher income countries having half the number of diagnosed patients as middle-income countries with practically the same number of machines available for delivering service in each grouping. In countries such as Antigua and Barbuda, St. Kitts and Nevis, Grenada, and St. Vincent and the Grenadines, the population size is less than the recommended quantum of one megavoltage machine per 180 000 inhabitants and thus, individually, will not result in the full utility of these machines. Therefore, satellite facilities must be considered as referral centers for treatment. Currently, unofficial arrangements exist among some of the island nations. These must be further explored and formalized for CARICOM and other Caribbean countries. Countries with existing collaborative bodies in place, such as the Organization of Eastern Caribbean States (OECS), are in an excellent position to draw on these partnerships. Therefore, services can be provided to other OECS nations which would serve the dual purpose of meeting the other countries’ needs and fully utilizing the machine. The collective population for Antigua and Barbuda, Dominica, Grenada, St. Vincent and the Grenadines, and St. Kitts and Nevis is approximately 441 000. Based on the IAEA population-based recommendations, their needs can be served by two external beam machines (2.5 machines). In the first instance, given that Antigua and Barbuda already has a functional machine, this serves as an appropriate starting point to discuss cost-effective use of existing services. Secondly, with the absence of high-dose rate brachytherapy in all of those islands, discussions surrounding its addition to the needed suite of services must be entertained while exploring options for additional services. The overarching challenge here is that, given the data presented, the majority of the other countries listed which do have machines have their own inherent staffing and equipment deficiencies. Therefore, offering services to additional countries in their current crisis serves only to strain their own resources. This is one of the reasons why Spence et al. suggested the establishment of international partnerships with developed countries and support from non-governmental groups and organizations [30]. In some other LMIC, international training partnerships have been undertaken. One such example is that between Cambodia and France to facilitate specialist training in the field [12].

The study was limited in that data was captured from one primary representative across institutions and not by multiple persons from the same institution. The institutions identified were not large; thus, it was presumed that little variation would have existed among potential responders. However, multiple responders within the institutions could have allowed for better cross-checking of data. Only Caribbean Community full member states were included in the study, which limits the potential for observations, which could then be generalized to the wider Caribbean. One strength of the study is the inclusion of almost all the Caribbean Community with full member status. The paper also comments on brachytherapy services, which is often missed in analyses on radiotherapy services where the focus often lies on external beam radiotherapy machines only. Given the incidence of cervical cancer in this region, its exclusion should be avoided. GLOBOCAN data was not available for some of the Caribbean countries, and thus, the data presented for these nations may over- or underestimate the actual reality. Apart from the preceding, equipment, and staffing needs estimated based on IAEA’s per population recommendations may further overestimate exact needs since age-standardized and crude cancer incidence rates in Caribbean countries can be less than that noted in other areas. IAEA patient-based criteria were thus included in the study analysis for comparison to per population recommendations [8]. Population data is likely more reliable; however, cancer is not a notifiable disease in many of these Caribbean countries and thus may be under-reported. Also, the GLOBOCAN website cautions interpretation of their data, which it admits may be limited, especially for LMIC [7]. The estimations made using this data may thus be modest.

Thus, in spite of cases where staffing surpluses are suggested with these figures, this may not be an exact representation. Additionally, actual utilization rates may be less than the suggested optimal rates (even in developed countries), and thus, the numbers recommended are only relevant in an ideal scenario where all patients needing radiation treatment are referred for and amenable to the same. Data on actual utilization rates for the region would provide a more appropriate reference for calculating current needs but are not readily available.

Overall, multiple barriers have been identified, which affect the implementation of high-quality radiotherapy services. They include a lack of funding to procure equipment, trained staff of all categories to operate the equipment (and programs to facilitate the same), infrastructure support for the service plus reliable and up-to-date national/regional data to inform decisions, and guide planning [3, 4, 31]. Radiotherapy must also be supplemented by access to appropriate medication (such as chemotherapy), equipped laboratories, skilled medical multi-disciplinary clinical team members (surgeons, pathologists, radiologists, etc.), and nursing support, to name a few. These, while not within the immediate scope of the current study, do bear mention as they form an integral part of holistic care.

However, focusing only on therapeutic intervention will only attack the existing problem. More programs need to be geared towards prevention and early detection. In some cases, early detection avoids the need for multi-modality treatment sparing the use of chemotherapy or radiation therapy and limiting treatment to surgical options only. Preventative campaigns and the use of vaccines, such as those used in the management of cervical cancer, have already been shown to decrease incidence rates [32]. Highlighting the staffing deficits which were detected in the study provides necessary information for governments to aid workforce planning. This is important given that appropriate funding needs to be allocated for training to meet the demands of the need which exists. It also provides critical guidance for countries that need to invest in procuring equipment to provide the necessary service. Most importantly, it highlights to policymakers the need for accurate and recent data on cancer incidence, mortality, and prevalence statistics as this information forms the basis from which projections can be made.

## Conclusions

CARICOM has significant challenges concerning the adequacy of therapeutic radiation services. Overall, only 43% of the CARICOM nations studied met at least one criterion for adequacy of equipment and staffing. However, the oncologists and medical physicists in these institutions often serve dual roles or offer services to other centers as part of their scope of practice. Only Antigua and Barbuda has met staff and equipment requirements for both patient and population-based criteria. Training programs are limited or, in some cases, such as the case of radiation oncology, are not available loco-regionally.

Additionally, staff retention is an ongoing problem. Pooled resources through a strategic all-inclusive Caribbean comprehensive action plan may be the ideal vehicle to engender change and optimize access but requires discussion and structured collaboration. It, however, should only serve as an interim plan given that most countries have insufficient resources internally, and capacity building is required for real advancement. The 2013 World Health Organization Global Action Plan for non-communicable diseases has committed member states to a target of 80% availability of the affordable, necessary technologies and essential medicines required to treat major non-communicable diseases including cancer [33]. While the region is not yet poised to meet the target, with continued collaboration, this may 1 day soon become a reality.

## Data Availability

All datasets used and/or analyzed during the current study are available from the corresponding author on reasonable request.

## Abbreviations

RT: Radiation therapy
CARICOM: Caribbean Community
IAEA: International Atomic Energy Agency
RTT: Radiation therapist
GLOBOCAN: Global Cancer Observatory
DIRAC: Directory of Radiotherapy Centers
N/A: Data not available
NRM: No radiotherapy machine
N/E: Not evaluable (where there was a gap in the data)
OECS: Organization of Eastern Caribbean States
LMIC: Lower- and middle-income countries
